# A framework for Controlled Human Infection Model (CHIM) studies in Malawi: Report of a Wellcome Trust workshop on CHIM in Low Income Countries held in Blantyre, Malawi

**DOI:** 10.12688/wellcomeopenres.12256.1

**Published:** 2017-08-24

**Authors:** Stephen B Gordon, Jamie Rylance, Amy Luck, Kondwani Jambo, Daniela M Ferreira, Lucinda Manda-Taylor, Philip Bejon, Bagrey Ngwira, Katherine Littler, Zoe Seager, Malick Gibani, Markus Gmeiner, Meta Roestenberg, Yohannie Mlombe, Wellcome Trust CHIM workshop participants

**Affiliations:** 1 The Malawi Liverpool Wellcome Trust Clinical Research Programme, Blantyre, Malawi; 2 Vaccines, Wellcome Trust, London, NW1 2BE, UK; 3 Liverpool School of Tropical Medicine, Liverpool, L2 5QA, UK; 4 The University of Malawi College of Medicine, Blantyre, Malawi; 5 KEMRI-Wellcome Trust Research Programme, Kilifi, Kenya; 6 Medicines and Poisons Board, Lilongwe, Malawi; 7 Policy, Wellcome Trust, London, NW1 2BE, UK; 8 Oxford Vaccines Group, Department of Paediatrics, Children’s Hospital, Oxford, OX3 9DU, UK; 9 Leiden University Medical Center, Leiden, 2333 ZA , Netherlands; 10 College of Medicine Research Ethics Committee, John Chiphangwi Learning Resource Centre, Blantyre, Malawi

**Keywords:** Controlled human infection model, CHIM, vaccine, workshop report

## Abstract

Controlled human infection model (CHIM) studies have pivotal importance in vaccine development, being useful for proof of concept, pathogenesis, down-selection and immunogenicity studies.  To date, however, they have seldom been carried out in low and middle income countries (LMIC), which is where the greatest burden of vaccine preventable illness is found.  This workshop discussed the benefits and barriers to CHIM studies in Malawi.  Benefits include improved vaccine effectiveness and host country capacity development in clinical, laboratory and governance domains.  Barriers include acceptability, safety and regulatory issues. The report suggests a framework by which ethical, laboratory, scientific and governance issues may be addressed by investigators considering or planning CHIM in LMIC.

## Introduction

Controlled human infection models (CHIM), using ICH GCP guidelines ^1^, have the potential to rapidly advance the development of vaccines with public health relevance to Africa. In a CHIM study, a well characterised strain of an infectious agent is administered at a controlled dose and by a specific route to carefully selected adult volunteers. Volunteers are closely monitored for evidence of carriage or infection under medical supervision to anticipate or manage symptoms of disease ^2^.

Modern CHIM studies undergo detailed independent review and oversight, and therefore are entirely unrelated to the unacceptable and unregulated infectious challenges carried out in the past. This article relates only to well-regulated CHIM studies in the last 70 years, in which more than 22,000 people have volunteered. These studies have examined the pathogenesis, clinical features, microbiology, and the immune response to more than 15 pathogens of public health importance, as shown in Table 1. This has led to important discoveries in host-pathogen dynamics, and has been used to make drug development pathways more efficient and less costly.

**Table 1.** A selection of recent CHIM studies, time periods and degree of utilization (2015).

Challenge model	Time periods of utilization	Number of challenge strains used so far	Approximate numbers of volunteers
**Typhoid &** **Paratyphoid** **(enteric fevers)**	1952–1974; 2012–present	2	2,200
**Influenza**	1976–present	4	1,500
**Malaria**	1986–present	4	1,000
**Pneumococcal** **carriage**	2000–2005; 2012–present	2	850
**Shigella**	1946–1995; 2006–present	9	750
**Enterotoxigenic** **Escherichia coli**	1971–1999; 2007–present	11	500
**Neisseria** **lactamica**		1	398
**Campylobacter**	1988–1998; 2009–present	5	225
**Norovirus**	1990–2005; 2012–present	3	220
**Cholera**	1983–2005; 2010–present	3	200
**Cryptosporidium**	1995–2006; 2014–present	5	110
**Respiratory** **Syncytial Virus** **(RSV)**	1985–2000; 2010–present	2	150
**Dengue**	1945–1952; 2010–present	7	50
**Mycobacteria** **(BCG)**	2012–present	1	40

References for many of these studies are included in the comprehensive review by Darton (2015) ^5^.

To test host-pathogen interaction or vaccine targets in the country where the diseases occurs seems logical from ethical/social point of view because the potential benefits of the results are in those countries ^3^. But additionally, and equally very importantly, are the biological variables that might be important in host-pathogen or host-vaccine interaction, which are very different in LMICs compared to, for example, the UK. Genetics, infectious disease history, co-infections, immune status, and environmental factors might likely play a role in how the host responds to the pathogen and/or the vaccine, and can only be appropriately tested in the targeted settings. That rationale actually requires the model to be run in the LMIC countries where the diseases occur.

To date, no CHIM studies have been carried out in Malawi ^4^. This country has one of the lowest GDP in the world, with high burdens of morbidity and mortality from infectious disease. The reasons why CHIM studies are not carried out in Low and Middle Income Countries (LMIC) include technical, clinical, ethical and regulatory issues, as well as cultural norms. The cost-benefit analysis of CHIM studies might, however, show substantial benefit to LMIC, as an expedient approach to major infectious challenges in a resource poor setting. A workshop was convened to discuss the scientific and public health value of conducting CHIM studies in Malawi, and the research governance issues that need to be addressed. There was a plurality of views from workshop participants, specifically around the readiness of scientific and clinical stakeholders in Malawi, and the advisability of challenge model implementation. These are recognised in Table 2, with key challenges highlighted below. Where these are addressed, we propose a possible roadmap for undertaking CHIM in Malawi.

**Table 2.** Issues highlighted for any potential CHIM studies in Malawi.

Potential benefits	Challenges
**Accelerating or streamlining vaccines/treatment** relevant to the national health burden. **Building local capacity** in: clinical facilities; laboratory diagnostics; experimental medicine; clinical governance; and regulatory confidence. **Opportunity to construct Malawi’s regulatory framework to** **suit its own needs** rather than adopting one from the EU/US. **Improving science capacity** through work-based training and mentorship of local scientists. **Understanding key scientific questions relevant to public** **health** in Malawi, including the effect of genetics, endemic infectious disease and co-infections, immune status, and environmental factors. These unique combinations of effects cannot be correctly understood in a model run elsewhere.	**Current infrastructure and clinical facilities may not be** **ready** for CHIM (specifically, monitoring and supporting adverse events e.g. on intensive care units). **Inherent vulnerabilities may hamper fully informed consent** in the local context (languages, assessment understanding, participant criteria suitability, cultural family/group consenting). **Poor community hygiene and sanitation infrastructure** **could prevent effective control measures** (e.g. typhoid in out- patient settings). **Production of challenge stain locally may have QA issues,** **but “international” strains may be less relevant** - resulting long supply chains need careful management. **Consensus on appropriate compensation for monetary** **and opportunity costs is lacking:** the balance between appropriate recompense and incentivisation is difficult and locally variable. **Malawi has not yet hosted Phase 1 studies**, or those including healthy volunteers who are expected to become symptomatic due to the research protocol; there is a relative shortage of skills and experience

## CHIM design issues

Considerations in the design, recruitment, and microbiology of CHIM studies have been recently reviewed ^5^. CHIM studies provide the opportunity to study many aspects of the course of a microbial infection, along with assessing the response to treatment and the efficacy of naturally acquired and vaccine induced immune responses. They provide an efficient framework for selecting vaccine and drug candidates for further development and can be carried out on a much smaller number of volunteers, and much more rapidly than Phase IIb-III studies. Protection against specific microbial strains can be evaluated in a controlled setting, and biomarkers to resistance and protection against infection can be efficiently assessed. Therefore, CHIM studies can accelerate vaccine development and evaluation.

CHIM studies require safe and accurate microbiology, good clinical facilities, careful recruitment and monitoring and close governance. Models are limited to those pathogens that are both detectable and treatable or self-limiting; complete clearance is usually required before the end of the study. Attenuated strains are sometimes used, to minimize potential symptoms and risks, or to assess the challenge agent itself as a vaccine candidate.

## Current CHIM models in LMIC

CHIM have been developed for many diseases where the global burden is predominantly borne by LMIC, but few have been carried out among the populations of these countries. Malaria infection models have a very long history in Africa ^6^, and has recently been revived in Kenya ^7^, Tanzania ^8^ and Gabon ^9^. A *Shigella sonnei* CHIM study in Thailand, as well as a cholera study, were the first enteric infection CHIM studies in LMIC ^10^.

## Reasons to consider further CHIM in LMIC

Vaccine trial results have been highly variable in different global regions, and sometimes promising results from high income settings have not been replicated in LMIC. There are good reasons to expect that, compared with high income countries, there are different host-pathogen relationships in LMIC for vector-borne diseases (malaria and dengue), enteric diseases (cholera, enterotoxigenic *Escherichia coli* [ETEC], *Shigella*, *Campylobacter*, typhoid fevers and norovirus), and some respiratory pathogens (influenza, RSV, pneumococcus, *Neisseria* and non-tuberculous mycobacteria). These differences in disease epidemiology are a result of multiple factors, including naturally acquired immunity, dietary factors, intestinal microbiota, and the genetic profile of the host population.

New vaccines against several of the diseases for which CHIM have been developed, are either licensed or in intermediate or advanced stages of development. These diseases include malaria, cholera, ETEC, *Shigella*, typhoid, influenza, pneumococcal pneumonia and TB. There is, however, no affordable and achievable path to licensure for most of these candidate vaccines and no means of choosing between the vaccine candidates based on the immunological response of vaccinated individuals in exposed population.

CHIM have played a pivotal role in the development of vaccines against enteric diseases including the cholera vaccine Dukoral® and typhoid vaccine Ty21a (Vivotif®) ^11, 12^. Additionally, in 2016, a live, oral, cholera vaccine (Vaxchora®) was the first vaccine where CHIM provided the primary evidence of effectiveness required for licensure by the FDA for use in the US. The path to licensure for new malaria and typhoid vaccines now includes CHIM at Phase 2a ^13^. The development of current malaria vaccine RTS,S (GSK) was facilitated by CHIM ^14^.

In the case of pneumococcal vaccine development, carriage is a tenable surrogate endpoint in the testing of new vaccines, as the number of subjects required for a clinical Phase 3 study including current conjugate vaccine in the control arm exceeds 200,000 individuals. CHIM studies are typically much smaller, requiring fewer than 150 subjects, using carriage as an endpoint ^15^. These may provide data to discontinue development of under-performing vaccine candidates, particularly when combined with infant natural carriage studies, and enable the identification of a vaccine to take forward.

Scientific reasons to consider further CHIM in LMIC therefore include: the need to focus testing of late stage novel vaccines; the record of CHIM success in enteric vaccine and malaria vaccine development; the potential for accelerated development of as yet undiscovered vaccines.

## Considerations in designing CHIM studies specific to LMIC

As CHIM studies involve the deliberate infection of healthy volunteers with infectious agents, these studies are technically challenging, and require careful ethical consideration of both risk to participants, and to their contacts, and the provision of robust clinical service to support the participant should an adverse event occur. These key issues of acceptable levels of risk and adequately informed consent are paramount in any global region ^5^, with specific issues which make LMIC more vulnerable. As an overarching principle, CHIM studies in LMIC should be designed with a “equivalent international” standard, in which the study is conducted at least as well as it could have been done anywhere in the world, exemplified by leading LMIC work on malaria studies ^7^.

### Clinical facility & healthcare provision considerations

Clinical facilities to recruit and manage study participants in LMIC must ensure that the infection risk is no greater than elsewhere in the world. This consideration must extend to include not only the clinical rooms, but also referral pathways, transport, diagnostic investigation and treatment. The design and development of CHIM studies in LMIC under the equivalent international standard may provide an opportunity to improve clinical services in resource-poor settings.

The decision to adopt inpatient or outpatient design, including consideration of isolation, must be made based on the pathogen-associated risk in the local context. Clinical assessment will typically be used at screening to identify healthy participants at minimal risk of incidental illness during the CHIM. Inclusion and exclusion criteria for participants in endemic settings may include recent and past infection, determined by available medical records and immunological screening. Further, in endemic settings, consideration of circulating vectors (e.g. malaria), community prevalence of carriage (e.g. pneumococcal carriage) and the level of community hygiene and sanitation infrastructure (e.g. typhoid) to ensure community safety are required.

### Technical considerations

*Challenge strain –* the choice and availability of a pathogen strain is a crucial methodological consideration. Quality Assurance (QA) of inocula is key, from manufacture (which may require Good Manufacturing Practice certification) through to a regulated delivery pathway to the volunteer. In the LMIC context, QA considerations from production to administration are a particular logistic challenge. While imported stock will have a long supply line, a centrally manufactured product will maximise comparability between sites. For locally generated stock, GMP production standards may be more challenging, but strict QA procedures are required at the study site whichever route is taken.

*Endpoints –* Endpoints for CHIM studies must be consistently defined, and easily identified before the onset of any significant pathology, in order to minimise the risk to participants, and to ensure that clinical or scientific outcomes are assessed. Endpoints vary across models, but can be dependent on the diagnosis of infection, the development of infection symptoms, or the assessment of infection severity. Using endpoints of the development of infection provides a unique opportunity to identify and validate novel biomarkers and diagnostic tests. In the LMIC context, CHIM endpoint diagnosis could provide valuable capacity building within local laboratory services.

### Community perception and engagement

Community perceptions vary widely around the world with respect to the understanding of infection risk, disease severity, treatment availability and effectiveness and vaccination to prevent disease ^7^. The underlying anxieties are likely to be similar in many regions, but the means to address them should be locally determined in LMIC in advance of study design, using regionally appropriate programmes of community engagement, consultation and education. It is recommended that CHIM studies are based on a deep understanding of the local community’s perceptions based on prior experience and previous engagement, and from a position of established mutual trust.

### Ethical considerations

Volunteer recruitment, advertising and the means to ensure that consent is fully informed will vary in different regions ^16^. The international standards for consent do not vary, but the means of information provision and of gaining a response are different in many LMIC. Proper methods must be developed by consultation with communities and with locally experienced researchers. Concepts of infection and disease may vary widely as may the extent of individual autonomy in providing consent. Advertising and remuneration are also different between regions and appropriate stakeholder consultation should determine the balance between incentivised research (too much perceived benefit) and lack of appropriate compensation for the negative impact on earnings and opportunity cost for participants (too little remuneration). A full study of these considerations has been reported from Kenya ^7^. Research Ethics Committees (RECs) have developed regulatory and ethical review and oversight experience with CHIM studies over time. At an early stage of development, local RECs should be encouraged to develop relationships with CHIM-experienced RECs to seek advice and share experiences. Training materials could be developed by collaboration between research sites and groups.

### Legal and regulatory considerations

CHIM studies have a more complex regulatory requirement in the US than the European Union (including the UK). In the US, but not in the EU, challenge and re-challenge studies that do not include a vaccine are subject to review at the national level. Further, in the US, the challenge strains themselves are usually required to meet a level of compliance with GMP, similar to that required for vaccines entering Phase 1 clinical trials. In the EU/UK, this condition is commonly not required, as summarised in Table 3, although QA/QC procedures following GMP principles should be in place. In the UK, there are few guidelines for setting up new CHIM studies and a more structured pathway would be useful.

**Table 3.** Types of controlled human challenge studies and their regulatory requirements.

Study type and description	Regulatory requirements in the US	Regulatory requirements in the UK
**Challenge study**: A challenge strain of a pathogen is administered to healthy adult volunteers.	Study must be conducted under an Investigational New Drug (IND) application that has been approved by the US Food and Drug Administration. Challenge strains are ideally manufactured in compliance with GMP at a level similar to that required for a product to be studied in a Phase 1 clinical trial. Once approved, INDs (or master drug files) for challenge strains may be cross-referenced in subsequent studies. In addition, the study must undergo review and approval by an IRB, or multiple IRBs if the study is to be conducted at multiple sites. Other review agencies may also have jurisdiction under certain circumstances (see Table 2).	Study does not require review by the MHRA if it does not involve a vaccine or other IMP. The challenge strains do not necessarily need to meet GMP requirements. Review and approval by an IRB is a requirement, and by other agencies in select circumstances (see Table 2).
**Re-challenge study**: A challenge strain of a pathogen is administered to healthy adult volunteers previously challenged with the same strain or a heterologous strain.		
**Vaccine trial using a challenge** **model**: A candidate vaccine is administered to healthy adult volunteers, who are later (or in some cases, previously) challenged with one or more strains of the pathogen against which the vaccine or drug is directed.	Candidate vaccine must be administered under an approved IND application and must undergo ethical review and approval by the relevant IRB(s), in addition to the approvals for the challenge strain and study procedures as described above.	Candidate vaccine must be administered under an MHRA approved application. The protocol must also undergo ethical review and approval by the relevant IRB(s).

Regulatory authorities in addition to RECs include medicines regulatory boards (e.g. Medicines and Healthcare products Regulatory Agency (MHRA, UK), European Medicines Agency (EMA); Food and Drug Administration (FDA, USA), Pharmacy, Medicines and Poisons Board (PMPB, Malawi). Legal positions vary substantially and may include the Departments of Agriculture and Environment. In the USA challenge agents are classed as pharmacologically active agents, and the FDA must approve their release. In contrast, in the UK challenge agents are not classed as pharmaceutical products and regulatory approvals from the MHRA are not required before CHIM experiments. In both the US and EU, CHIM studies that incorporate testing of an Investigational Medicinal Product (IMP) require the relevant medicines agency approval. The Kenyan regulatory process is managed by a REC, Pharmacy and Poisons Board, and local research governance processes.

### Sponsorship, governance and compensation

CHIM studies in LMIC have strong collaborative links with the research institution in which the CHIM was initially developed, although may be led from research institutions in the country. Sponsorship has been undertaken by the original institution, or in the case of IMPs, by the commercial entity responsible for developing the IMP to market. Data Monitoring and Safety Boards are recommended for all CHIM studies. These boards should be small, and include statistics, ethics, technical and clinical advisors that are immediately responsive to the study team and sponsor. Insurance requirements vary by country, but no-fault compensation would normally be preferred as for any clinical trial.

### Genetically modified organisms

Using strains that have been genetically modified or attenuated requires additional approvals in most regions (the release of ‘genetically modified’ organisms [GMO]). Strain libraries and large-scale production of standardised pre-frozen strains may make the regulation of GMO more straightforward as data obtained in each study site will inform other experiments. GMO-related regulatory requirements vary by country, but are often regulated by the Ministry of Agriculture (such as DEFRA in UK).

## Summary: A possible roadmap for CHIM studies in Malawi

### Consider the need, benefit, science, ethics and model quality

In relation to CHIM studies in Malawi, the first consideration should be whether the burden of disease being studied is of sufficient importance to justify the risks associated with participant involvement, and the costs, including opportunity costs, of the effort. This is most easily judged by the degree of alignment with the National Research Agenda: in a resource constrained setting, there is little justification for research outside the national strategy.

The second consideration is whether the scientific and ethical case to justify the work has been met. In the LMIC context, the science may differ as a result of endemic exposure to pathogen, vector and co-infections. The ethical issues are similar in high and low income settings, but have local contextual nuance as well as the “equivalent international standard” principle. Inoculum preparation, dose and delivery considerations will vary by pathogen, study and site.

Third, we consider that an important factor is whether introduction of the model would substantially increase the national capacity in clinical facilities, laboratory diagnostics, experimental medicine, clinical governance and regulatory confidence. The capacity building element is a major ethical consideration in the LMIC context, particularly if CHIM studies can materially improve the government health services.

**Figure 1.**
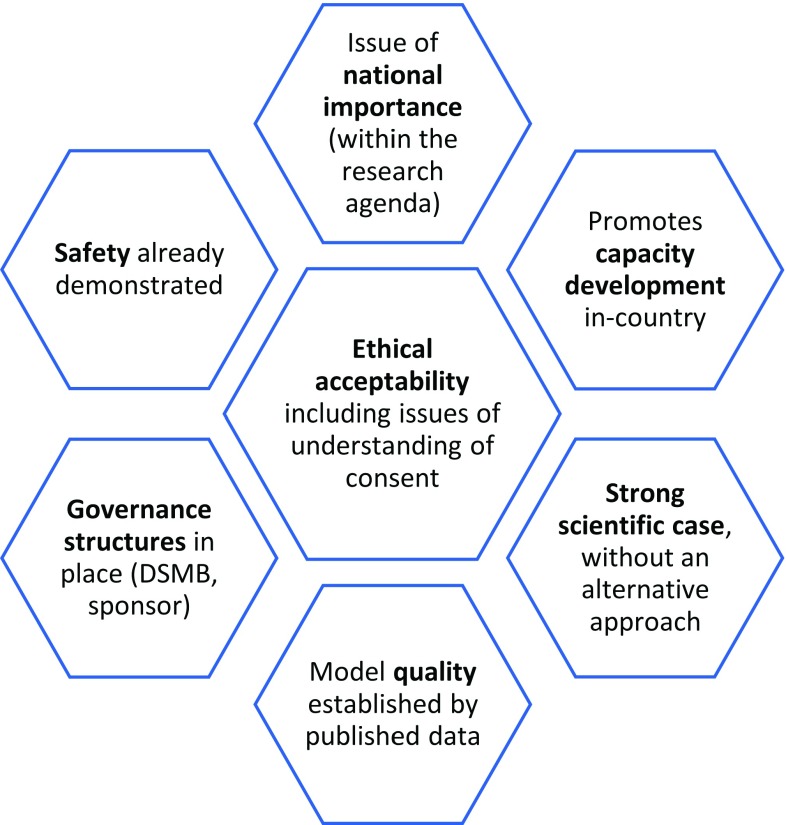
A framework for consideration of CHIM in Malawi.

Fourth, it is understood that, at this time, CHIM models should be developed in maximally resourced settings before introduction to LMIC. Before transition to LMIC, several studies should have arisen, from which the quality of the CHIM might be assessed. This set of considerations is laid out in Figure 1.

### Regulatory considerations

The WHO Expert Committee on Biological Standardization, 2016, ‘Human Challenge Trials for Vaccine Development: regulatory considerations’ ^17^ recognised that regulation of CHIM trials need to be well defined by the national regulatory authorities and vaccine developers, and manufacturers need to be aware of regulatory expectations.

In Malawi, the National Commission for Science and Technology (NCST) appoints the National Health Sciences Research Committee (NHSRC) to review the application to conduct any CHIM studies, as this type of research falls under the category of “national interest”. The PMPB is the drug regulatory authority that approves the use of drugs and vaccines. These authorities are the appropriate bodies to regulate CHIM. At this time, there is an opportunity for Malawi to define its own regulatory pathway for CHIM work.

## Conclusion

The workshop concluded that CHIM studies should be considered in Malawi if the studies addressed public health challenges and needs on the National Research Agenda, ensured capacity development, and had appropriate scientific and ethical rigour. This rigour would include the considerations of benefit and risk described for any CHIM, together with the local LMIC considerations laid out in this workshop report.
