# Supplementary material for: A framework for Controlled Human Infection Model (CHIM) studies in Malawi: Report of a Wellcome Trust workshop on CHIM in Low Income Countries held in Blantyre, Malawi
Source: Wellcome Open Res. 2017 Aug 24;2:70. [Version 1] doi: 10.12688/wellcomeopenres.12256.1 (PMC5627502; doi:10.12688/wellcomeopenres.12256.1)
Supplement: Peer review history

# Peer review: Referee response for version 1

**DOI:** 10.21956/wellcomeopenres.13269.r25340

**Published:** 2017-10-02

**Authors:** Cameron P. Simmons

**Affiliations:** 1 Department of Microbiology and Immunology, Peter Doherty Institute, University of Melbourne, Melbourne, Vic, Australia

This is generally a well-crafted synopsis of the regulatory, logistical, ethical and community issues facing the performance of CHIM research in LMIC. Some minor points for consideration.

1. The author state “ *Clinical facilities to recruit and manage study participants in LMIC must ensure that the infection risk is no greater than elsewhere in the world*.” Rephrase for greater clarity- is this a reference to risks faced by participants or risks posed to community contacts/environment?
2. *Challenge strain and method of production– * most LMIC will not be able to manufacture GMP stocks of challenge pathogen– we are then left with a loose framework (i.e. the UK scenario) for “home-made” brews of challenge strain production that accepts a pragmatic but lower level of scrutiny on production and testing. Should the authors be more forceful in motivating the scientific and regulatory community for guidance on this issue? i.e. what are the minimum requirements for production and testing?
3. Where relevant, insert a sentence or two to expand upon the differences between human infection models *versus* disease models.
4. The authors rightly point to the importance of deep and wide community engagement before and during CHIM establishment. It might be useful to describe how “acceptance” could be measured before during after CHIM research. e.g. via discussions with community reference groups and/or civil society actors.
5. Table 3. Could include mention of other applications of CHIM, e.g. prophylaxis or therapeutic studies of novel drug candidates.

I have read this submission. I believe that I have an appropriate level of expertise to confirm that it is of an acceptable scientific standard.

# Peer review: Referee response for version 1

**DOI:** 10.21956/wellcomeopenres.13269.r25335

**Published:** 2017-08-29

**Authors:** Patrick J. Lillie

**Affiliations:** 1 Hull and East Yorkshire Hospitals NHS Trust, Hull, UK

This is a clear description of the workshop on controlled human infection model (CHIM) in Malawi. As such there is no new data or experimental details to analyse / review and the article is scientifically sound, covering the background and available literature on CHIM studies in the discussion. Due to the paucity of CHIM studies in low / middle income countries (LMIC) it would have been good to have had a more in depth review of these studies in higher income countries, particularly those that are more relevant to LMIC conditions e.g. the malaria and typhoid challenge studies. The figures and tables are clear and the road map discussion is a good guide to the challenges in developing these studies (a comment in this section on volunteer recruitment and remuneration in LMIC as opposed to higher income would have been interesting).

I have read this submission. I believe that I have an appropriate level of expertise to confirm that it is of an acceptable scientific standard.
